# Raynaud’s phenomenon

**Published:** 2011-10

**Authors:** HD Solomons

**Affiliations:** Highlands North, South Africa

Raynaud’s phenomenon is a disorder, causing discolouration of the fingers. It is thought to be related to vasospasm. It can also affect the toes and other areas. The nails become brittle with longitudinal ridges. The condition was named after Maurice Raynaud (1834–1881).

The condition is the result of a vasospasm that decreases the blood supply to various organs. The triggers are cold and emotional stress. Raynaud’s disease is idiopathic and Raynaud’s syndrome is secondary to an initiating factor. One can measure the temperature of the hands to differentiate between the two forms. The primary form can progress to the secondary form. Complication in the secondary form is progression of the disorder to fingertip gangrene.

Raynaud’s phenomenon is an exaggerated response to emotional stress or cold. The sympathetic system causes severe vasoconstriction of the peripheral vessels and an axillary sympathectomy may well alleviate the disorder. The vasoconstriction leads to tissue hypoxia.

Chronic recurrent cases of Raynaud’s phenomenon can result in atrophy of the skin, subcutaneous tissue and muscle. Cases have been described where ischaemic gangrene and ulceration result. The disorder must be differentiated from acrocyanosis and pernio (chilblains).

There are specific medicines that are peripheral vasodilators, and the wearing of gloves may alleviate the disorder. The colour changes are usually from white to blue and then red. White occurs with the vasospasm, blue is due to cyanosis and red is from the resultant vasospasm related to prolonged vasoconstriction.

